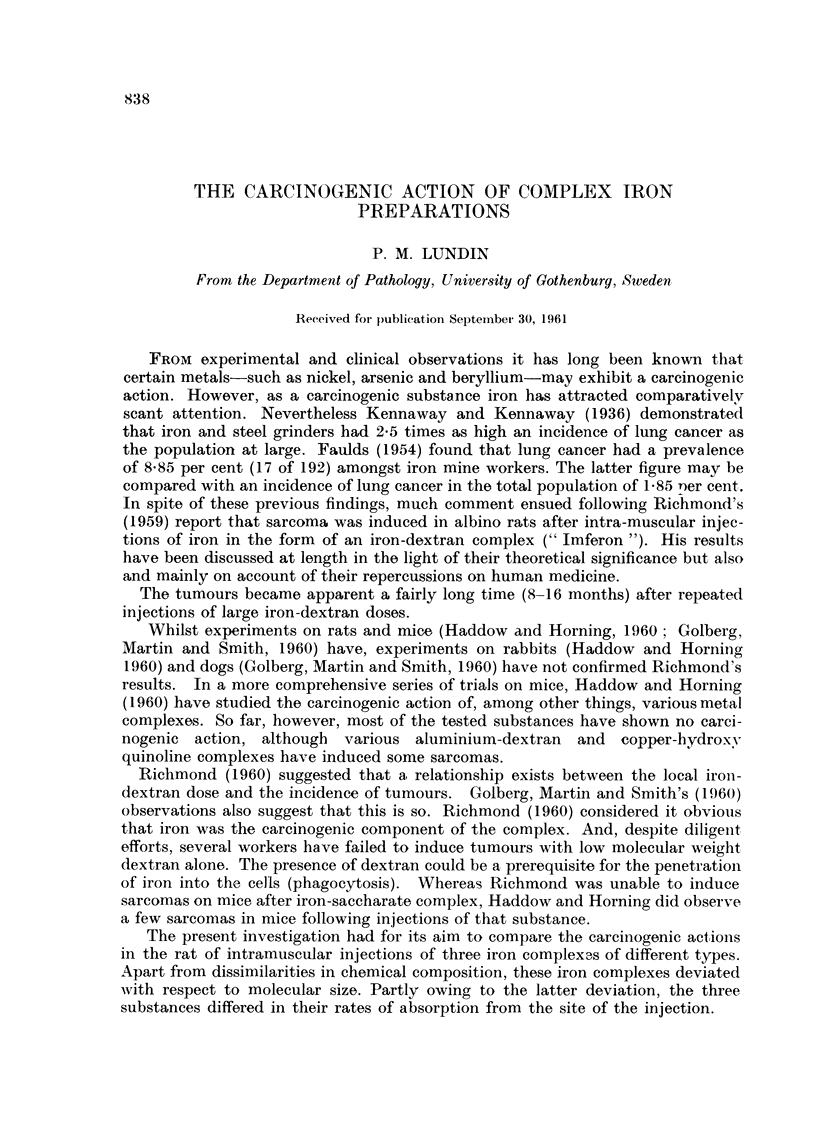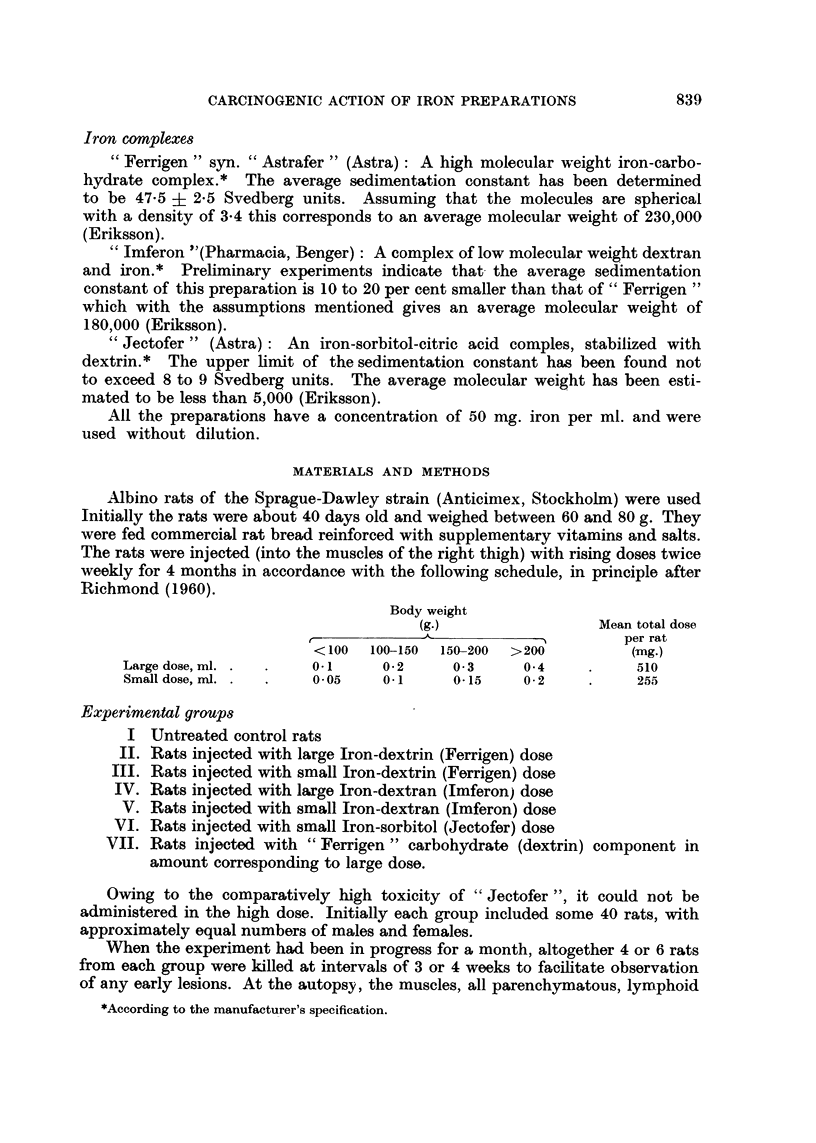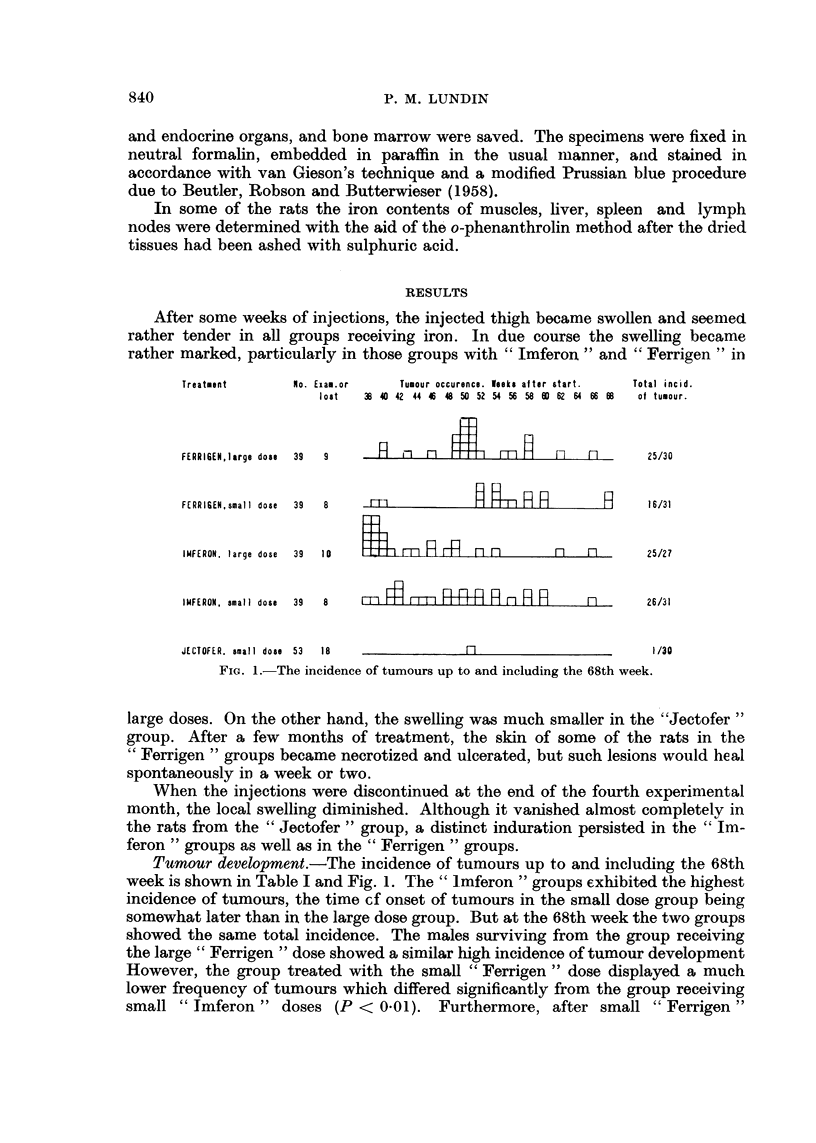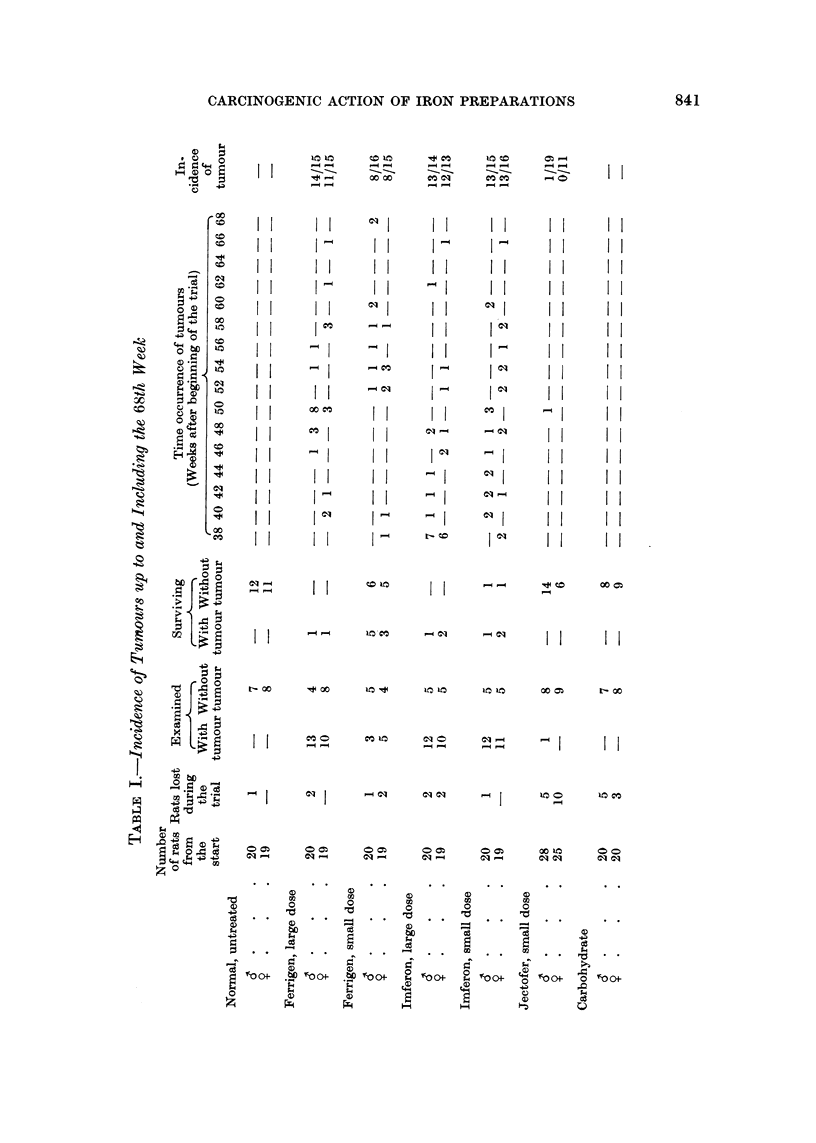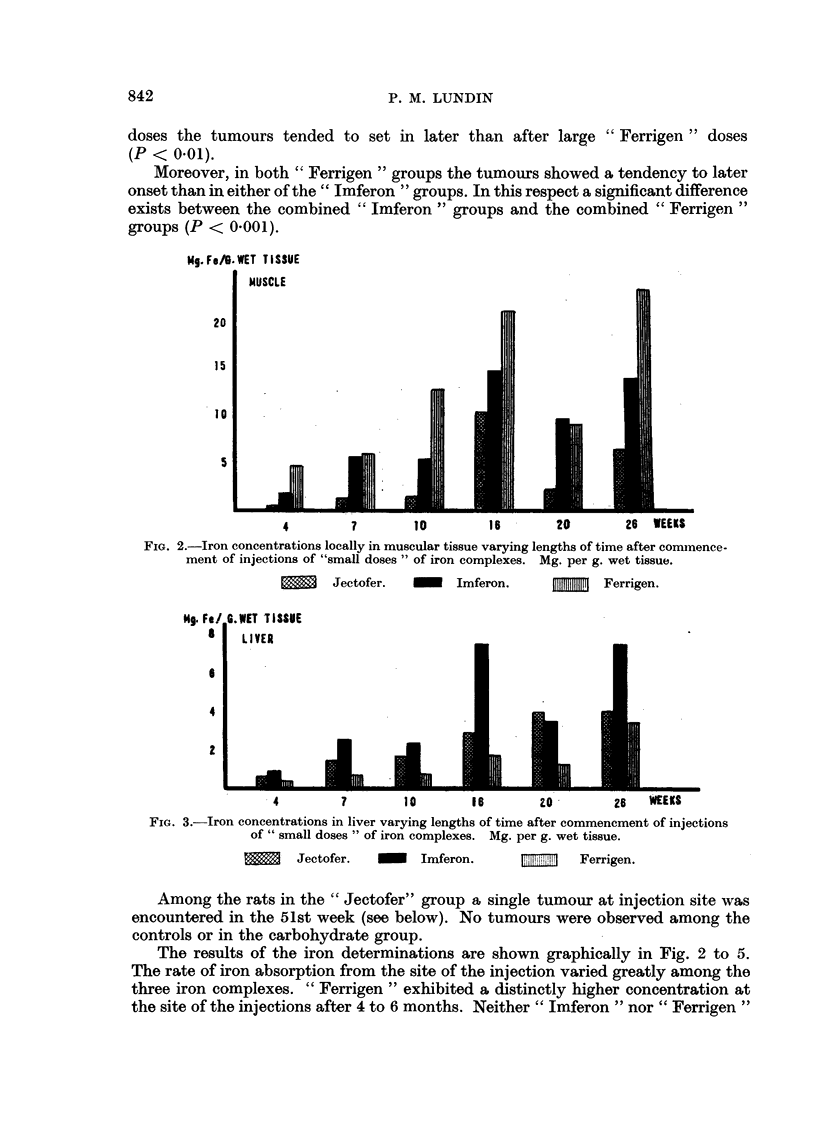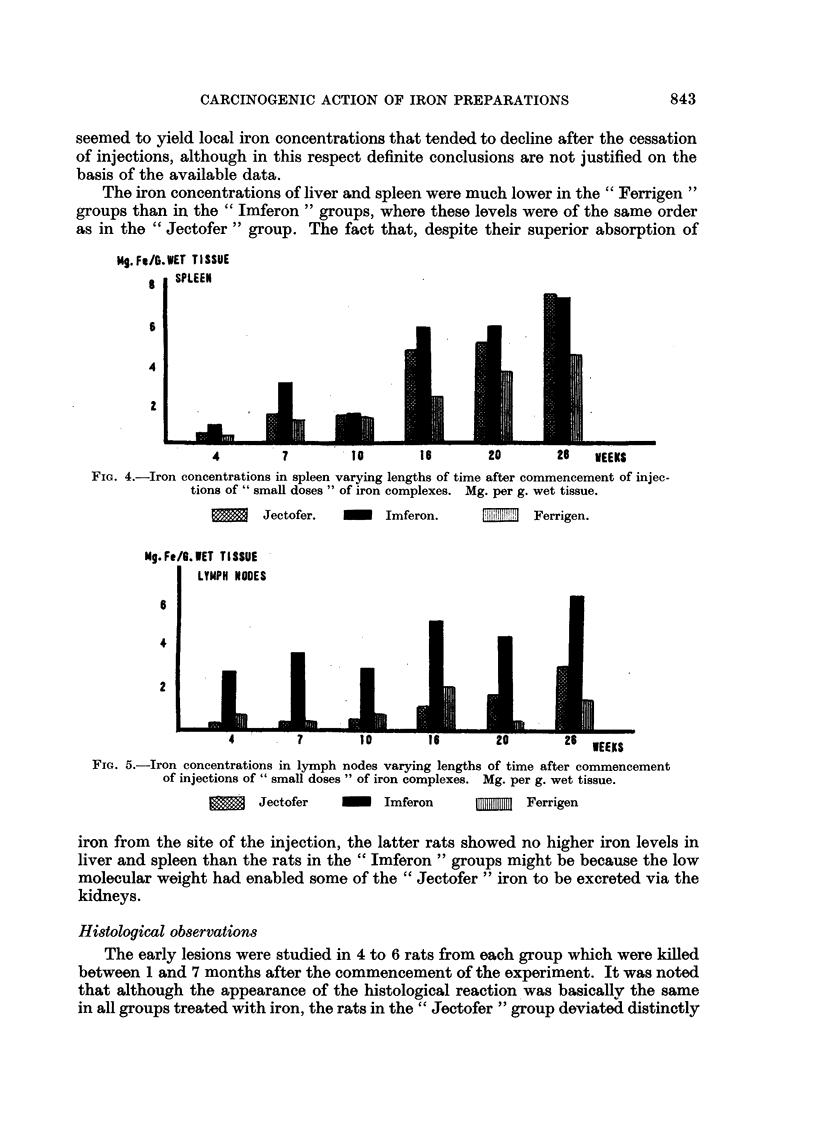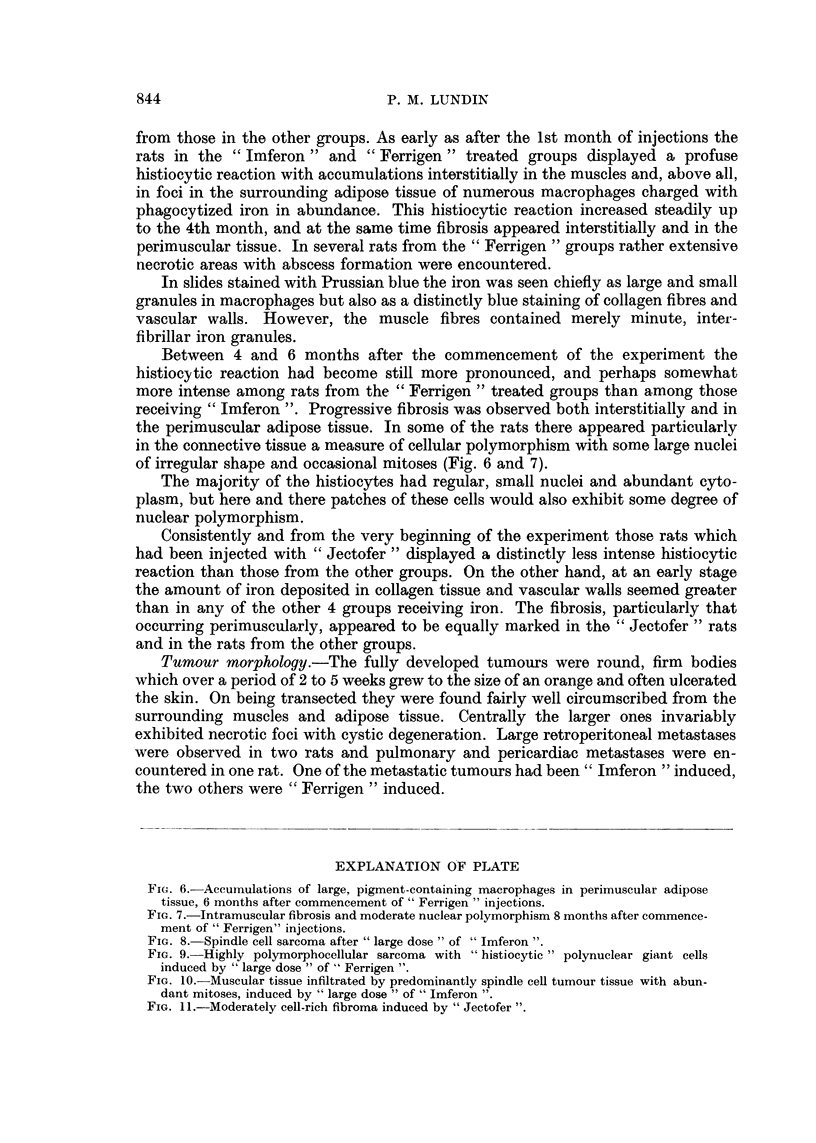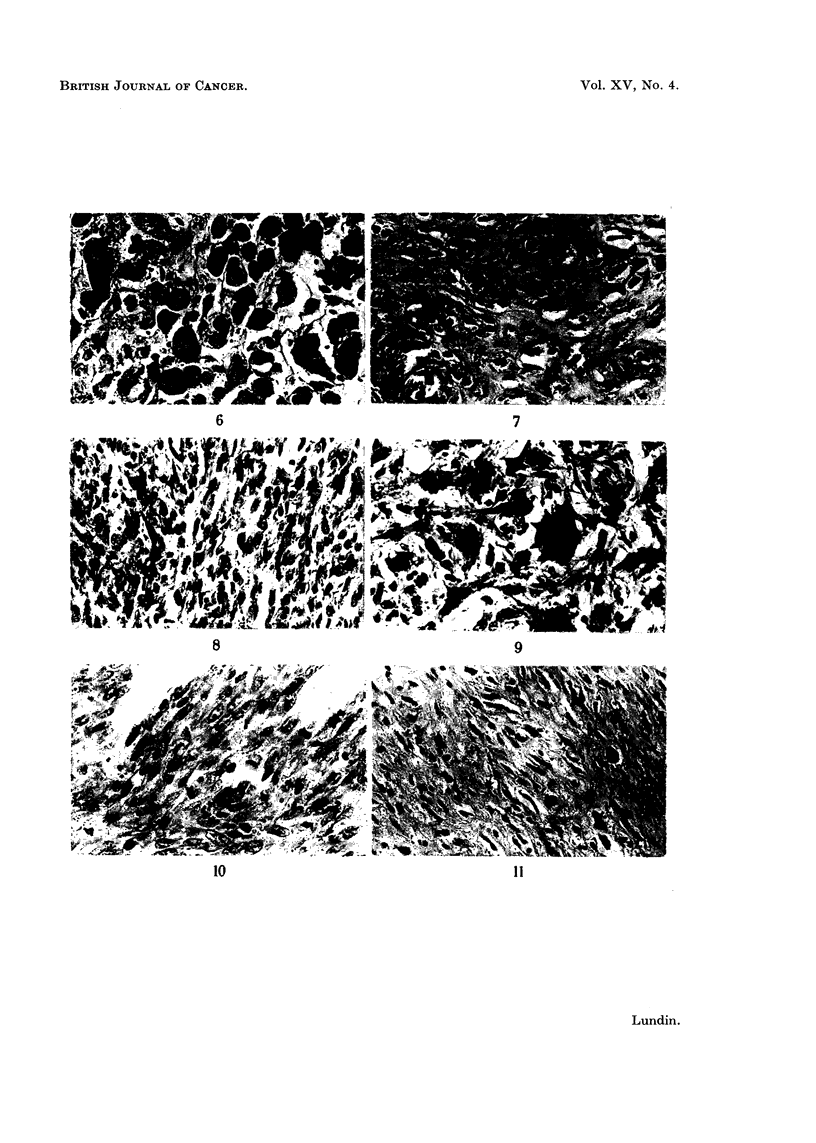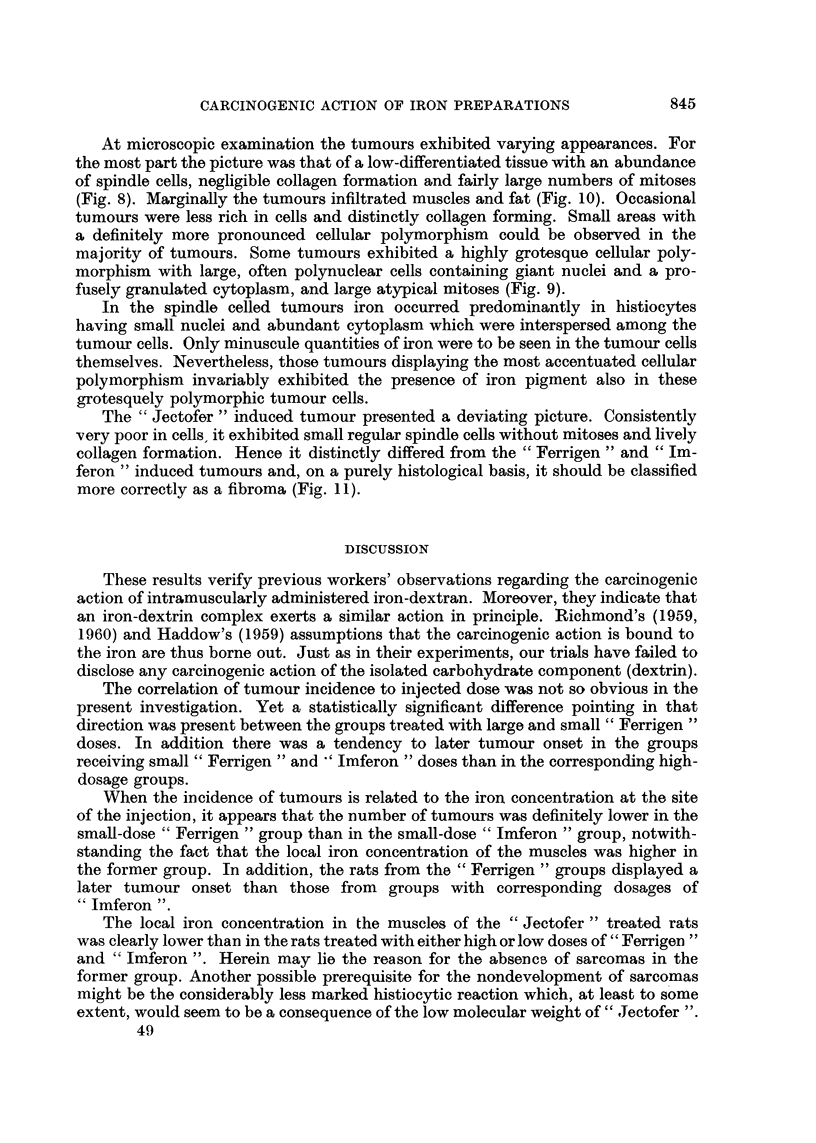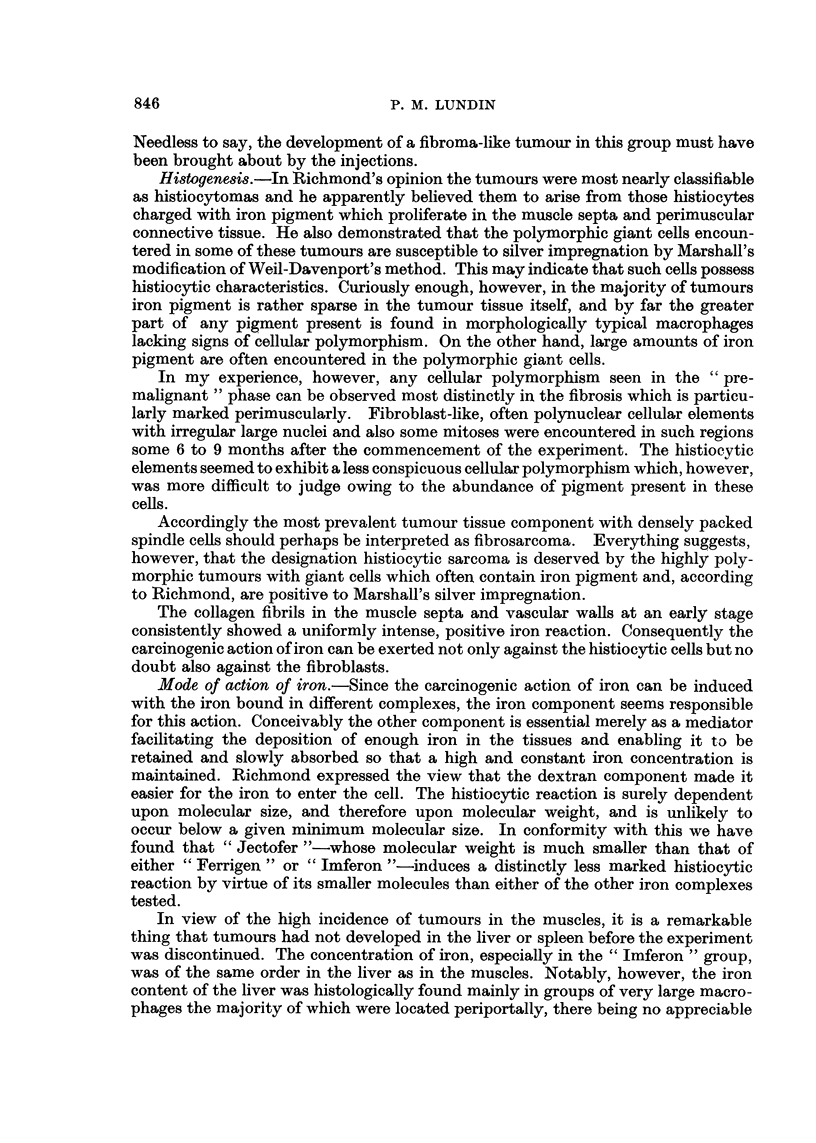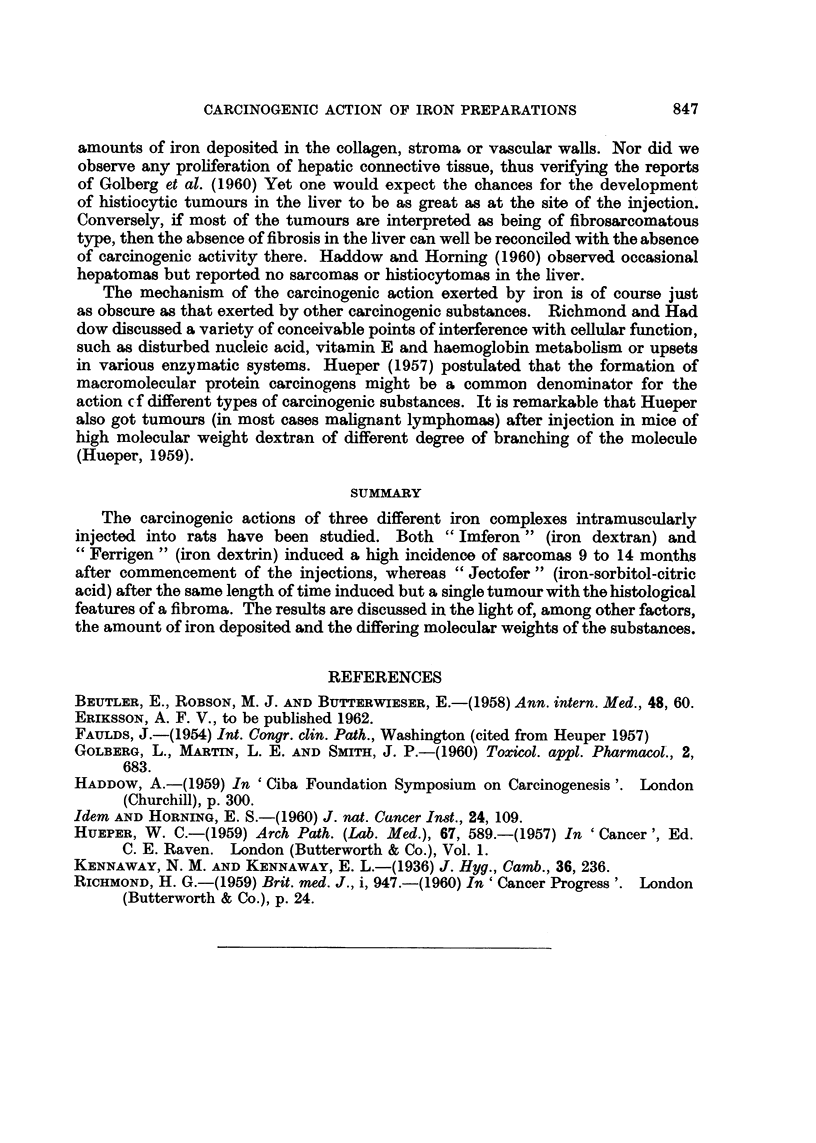# The Carcinogenic Action of Complex Iron Preparations

**DOI:** 10.1038/bjc.1961.97

**Published:** 1961-12

**Authors:** P. M. Lundin

## Abstract

**Images:**


### THE CARCINOGENIC ACTION OF COMPLEX IRON PREPARATIONS

P. M. LUNDIN

*From the Department of Pathology, University of Gothenburg, Sweden*

Received for publication September 30, 1961

FROM experimental and clinical observations it has long been known that certain metals—such as nickel, arsenic and beryllium—may exhibit a carcinogenic action. However, as a carcinogenic substance iron has attracted comparatively scant attention. Nevertheless Kennaway and Kennaway (1936) demonstrated that iron and steel grinders had 2·5 times as high an incidence of lung cancer as the population at large. Faulds (1954) found that lung cancer had a prevalence of 8·85 per cent (17 of 192) amongst iron mine workers. The latter figure may be compared with an incidence of lung cancer in the total population of 1·85 per cent. In spite of these previous findings, much comment ensued following Richmond's (1959) report that sarcoma was induced in albino rats after intra-muscular injections of iron in the form of an iron-dextran complex (" Imferon "). His results have been discussed at length in the light of their theoretical significance but also and mainly on account of their repercussions on human medicine.

The tumours became apparent a fairly long time (8–16 months) after repeated injections of large iron-dextran doses.

Whilst experiments on rats and mice (Haddow and Horning, 1960 ; Golberg, Martin and Smith, 1960) have, experiments on rabbits (Haddow and Horning 1960) and dogs (Golberg, Martin and Smith, 1960) have not confirmed Richmond's results. In a more comprehensive series of trials on mice, Haddow and Horning (1960) have studied the carcinogenic action of, among other things, various metal complexes. So far, however, most of the tested substances have shown no carcinogenic action, although various aluminium-dextran and copper-hydroxy quinoline complexes have induced some sarcomas.

Richmond (1960) suggested that a relationship exists between the local iron-dextran dose and the incidence of tumours. Golberg, Martin and Smith's (1960) observations also suggest that this is so. Richmond (1960) considered it obvious that iron was the carcinogenic component of the complex. And, despite diligent efforts, several workers have failed to induce tumours with low molecular weight dextran alone. The presence of dextran could be a prerequisite for the penetration of iron into the cells (phagocytosis). Whereas Richmond was unable to induce sarcomas on mice after iron-saccharate complex, Haddow and Horning did observe a few sarcomas in mice following injections of that substance.

The present investigation had for its aim to compare the carcinogenic actions in the rat of intramuscular injections of three iron complexes of different types. Apart from dissimilarities in chemical composition, these iron complexes deviated with respect to molecular size. Partly owing to the latter deviation, the three substances differed in their rates of absorption from the site of the injection.

*Iron complexes*

" Ferrigen " syn. " Astrafer " (Astra) : A high molecular weight iron-carbohydrate complex.* The average sedimentation constant has been determined to be 47·5 $\pm$ 2·5 Svedberg units. Assuming that the molecules are spherical with a density of 3·4 this corresponds to an average molecular weight of 230,000 (Eriksson).

" Imferon "(Pharmacia, Benger) : A complex of low molecular weight dextran and iron.* Preliminary experiments indicate that the average sedimentation constant of this preparation is 10 to 20 per cent smaller than that of " Ferrigen " which with the assumptions mentioned gives an average molecular weight of 180,000 (Eriksson).

" Jectofer " (Astra) : An iron-sorbitol-citric acid comples, stabilized with dextrin.* The upper limit of the sedimentation constant has been found not to exceed 8 to 9 Svedberg units. The average molecular weight has been estimated to be less than 5,000 (Eriksson).

All the preparations have a concentration of 50 mg. iron per ml. and were used without dilution.

### MATERIALS AND METHODS

Albino rats of the Sprague-Dawley strain (Anticimex, Stockholm) were used Initially the rats were about 40 days old and weighed between 60 and 80 g. They were fed commercial rat bread reinforced with supplementary vitamins and salts. The rats were injected (into the muscles of the right thigh) with rising doses twice weekly for 4 months in accordance with the following schedule, in principle after Richmond (1960).

| | Body weight (g.) | | | | Mean total dose per rat (mg.) |
|---|---|---|---|---|---|
| | <100 | 100–150 | 150–200 | >200 | |
| Large dose, ml. | 0·1 | 0·2 | 0·3 | 0·4 | 510 |
| Small dose, ml. | 0·05 | 0·1 | 0·15 | 0·2 | 255 |

*Experimental groups*

I Untreated control rats
II. Rats injected with large Iron-dextrin (Ferrigen) dose
III. Rats injected with small Iron-dextrin (Ferrigen) dose
IV. Rats injected with large Iron-dextran (Imferon) dose
V. Rats injected with small Iron-dextran (Imferon) dose
VI. Rats injected with small Iron-sorbitol (Jectofer) dose
VII. Rats injected with " Ferrigen " carbohydrate (dextrin) component in amount corresponding to large dose.

Owing to the comparatively high toxicity of " Jectofer ", it could not be administered in the high dose. Initially each group included some 40 rats, with approximately equal numbers of males and females.

When the experiment had been in progress for a month, altogether 4 or 6 rats from each group were killed at intervals of 3 or 4 weeks to facilitate observation of any early lesions. At the autopsy, the muscles, all parenchymatous, lymphoid

*According to the manufacturer's specification.

and endocrine organs, and bone marrow were saved. The specimens were fixed in neutral formalin, embedded in paraffin in the usual manner, and stained in accordance with van Gieson's technique and a modified Prussian blue procedure due to Beutler, Robson and Butterwieser (1958).

In some of the rats the iron contents of muscles, liver, spleen and lymph nodes were determined with the aid of the *o*-phenanthrolin method after the dried tissues had been ashed with sulphuric acid.

### RESULTS

After some weeks of injections, the injected thigh became swollen and seemed rather tender in all groups receiving iron. In due course the swelling became rather marked, particularly in those groups with " Imferon " and " Ferrigen " in

FIG. 1.—The incidence of tumours up to and including the 68th week.

large doses. On the other hand, the swelling was much smaller in the "Jectofer " group. After a few months of treatment, the skin of some of the rats in the " Ferrigen " groups became necrotized and ulcerated, but such lesions would heal spontaneously in a week or two.

When the injections were discontinued at the end of the fourth experimental month, the local swelling diminished. Although it vanished almost completely in the rats from the " Jectofer " group, a distinct induration persisted in the " Imferon " groups as well as in the " Ferrigen " groups.

*Tumour development.*—The incidence of tumours up to and including the 68th week is shown in Table I and Fig. 1. The " Imferon " groups exhibited the highest incidence of tumours, the time cf onset of tumours in the small dose group being somewhat later than in the large dose group. But at the 68th week the two groups showed the same total incidence. The males surviving from the group receiving the large " Ferrigen " dose showed a similar high incidence of tumour development However, the group treated with the small " Ferrigen " dose displayed a much lower frequency of tumours which differed significantly from the group receiving small " Imferon " doses ($P < 0{\cdot}01$). Furthermore, after small " Ferrigen "

TABLE I.—*Incidence of Tumours up to and Including the 68th Week*

| | Number of rats from the start | Rats lost during the trial | Examined: With tumour | Examined: Without tumour | Surviving: With tumour | Surviving: Without tumour | 38 | 40 | 42 | 44 | 46 | 48 | 50 | 52 | 54 | 56 | 58 | 60 | 62 | 64 | 66 | 68 | Incidence of tumour |
|---|---|---|---|---|---|---|---|---|---|---|---|---|---|---|---|---|---|---|---|---|---|---|---|
| Normal, untreated | | | | | | | | | | | | | | | | | | | | | | | |
| ♂ . . . | 20 | 1 | — | 7 | — | 12 | — | — | — | — | — | — | — | — | — | — | — | — | — | — | — | — | — |
| ♀ . . . | 19 | — | — | 8 | — | 11 | — | — | — | — | — | — | — | — | — | — | — | — | — | — | — | — | — |
| Ferrigen, large dose | | | | | | | | | | | | | | | | | | | | | | | |
| ♂ . . . | 20 | 2 | 13 | 4 | 1 | — | — | — | — | — | 1 | 3 | 8 | — | 1 | 1 | — | — | — | — | — | — | 14/15 |
| ♀ . . . | 19 | — | 10 | 8 | 1 | — | — | 2 | 1 | — | — | — | 3 | — | — | — | 3 | — | 1 | — | 1 | — | 11/15 |
| Ferrigen, small dose | | | | | | | | | | | | | | | | | | | | | | | |
| ♂ . . . | 20 | 1 | 3 | 5 | 5 | 6 | — | — | — | — | — | — | — | 1 | 1 | 1 | 1 | 2 | — | — | — | 2 | 8/16 |
| ♀ . . . | 19 | 2 | 5 | 4 | 3 | 5 | 1 | 1 | — | — | — | — | — | 2 | 3 | — | 1 | — | — | — | — | — | 8/15 |
| Imferon, large dose | | | | | | | | | | | | | | | | | | | | | | | |
| ♂ . . . | 20 | 2 | 12 | 5 | 1 | — | 7 | 1 | 1 | 1 | — | 2 | — | — | — | — | — | — | 1 | — | — | — | 13/14 |
| ♀ . . . | 19 | 2 | 10 | 5 | 2 | — | 6 | — | — | — | 2 | 1 | — | 1 | 1 | — | — | — | — | — | 1 | — | 12/13 |
| Imferon, small dose | | | | | | | | | | | | | | | | | | | | | | | |
| ♂ . . . | 20 | 1 | 12 | 5 | 1 | 1 | — | 2 | 2 | 2 | 1 | 1 | 3 | — | — | — | — | 2 | — | — | — | — | 13/15 |
| ♀ . . . | 19 | — | 11 | 5 | 2 | 1 | 2 | — | 1 | — | — | 2 | — | 2 | 2 | 1 | 2 | — | — | — | 1 | — | 13/16 |
| Jectofer, small dose | | | | | | | | | | | | | | | | | | | | | | | |
| ♂ . . . | 28 | 5 | 1 | 8 | — | 14 | — | — | — | — | — | — | 1 | — | — | — | — | — | — | — | — | — | 1/19 |
| ♀ . . . | 25 | 10 | — | 9 | — | 6 | — | — | — | — | — | — | — | — | — | — | — | — | — | — | — | — | 0/11 |
| Carbohydrate | | | | | | | | | | | | | | | | | | | | | | | |
| ♂ . . . | 20 | 5 | — | 7 | — | 8 | — | — | — | — | — | — | — | — | — | — | — | — | — | — | — | — | — |
| ♀ . . . | 20 | 3 | — | 8 | — | 9 | — | — | — | — | — | — | — | — | — | — | — | — | — | — | — | — | — |

Columns 38–68: Time occurrence of tumours (Weeks after beginning of the trial)

doses the tumours tended to set in later than after large " Ferrigen " doses ($P < 0{\cdot}01$).

Moreover, in both " Ferrigen " groups the tumours showed a tendency to later onset than in either of the " Imferon " groups. In this respect a significant difference exists between the combined " Imferon " groups and the combined " Ferrigen " groups ($P < 0{\cdot}001$).

FIG. 2.—Iron concentrations locally in muscular tissue varying lengths of time after commencement of injections of "small doses " of iron complexes. Mg. per g. wet tissue.

Jectofer. Imferon. Ferrigen.

FIG. 3.—Iron concentrations in liver varying lengths of time after commencment of injections of " small doses " of iron complexes. Mg. per g. wet tissue.

Jectofer. Imferon. Ferrigen.

Among the rats in the " Jectofer" group a single tumour at injection site was encountered in the 51st week (see below). No tumours were observed among the controls or in the carbohydrate group.

The results of the iron determinations are shown graphically in Fig. 2 to 5. The rate of iron absorption from the site of the injection varied greatly among the three iron complexes. " Ferrigen " exhibited a distinctly higher concentration at the site of the injections after 4 to 6 months. Neither " Imferon " nor " Ferrigen "

seemed to yield local iron concentrations that tended to decline after the cessation of injections, although in this respect definite conclusions are not justified on the basis of the available data.

The iron concentrations of liver and spleen were much lower in the " Ferrigen " groups than in the " Imferon " groups, where these levels were of the same order as in the " Jectofer " group. The fact that, despite their superior absorption of

FIG. 4.—Iron concentrations in spleen varying lengths of time after commencement of injections of " small doses " of iron complexes. Mg. per g. wet tissue.

Jectofer. Imferon. Ferrigen.

FIG. 5.—Iron concentrations in lymph nodes varying lengths of time after commencement of injections of " small doses " of iron complexes. Mg. per g. wet tissue.

Jectofer Imferon Ferrigen iron from the site of the injection, the latter rats showed no higher iron levels in liver and spleen than the rats in the " Imferon " groups might be because the low molecular weight had enabled some of the " Jectofer " iron to be excreted via the kidneys.

*Histological observations*

The early lesions were studied in 4 to 6 rats from each group which were killed between 1 and 7 months after the commencement of the experiment. It was noted that although the appearance of the histological reaction was basically the same in all groups treated with iron, the rats in the " Jectofer " group deviated distinctly from those in the other groups. As early as after the 1st month of injections the rats in the " Imferon " and " Ferrigen " treated groups displayed a profuse histiocytic reaction with accumulations interstitially in the muscles and, above all, in foci in the surrounding adipose tissue of numerous macrophages charged with phagocytized iron in abundance. This histiocytic reaction increased steadily up to the 4th month, and at the same time fibrosis appeared interstitially and in the perimuscular tissue. In several rats from the " Ferrigen " groups rather extensive necrotic areas with abscess formation were encountered.

In slides stained with Prussian blue the iron was seen chiefly as large and small granules in macrophages but also as a distinctly blue staining of collagen fibres and vascular walls. However, the muscle fibres contained merely minute, interfibrillar iron granules.

Between 4 and 6 months after the commencement of the experiment the histiocytic reaction had become still more pronounced, and perhaps somewhat more intense among rats from the " Ferrigen " treated groups than among those receiving " Imferon ". Progressive fibrosis was observed both interstitially and in the perimuscular adipose tissue. In some of the rats there appeared particularly in the connective tissue a measure of cellular polymorphism with some large nuclei of irregular shape and occasional mitoses (Fig. 6 and 7).

The majority of the histiocytes had regular, small nuclei and abundant cytoplasm, but here and there patches of these cells would also exhibit some degree of nuclear polymorphism.

Consistently and from the very beginning of the experiment those rats which had been injected with " Jectofer " displayed a distinctly less intense histiocytic reaction than those from the other groups. On the other hand, at an early stage the amount of iron deposited in collagen tissue and vascular walls seemed greater than in any of the other 4 groups receiving iron. The fibrosis, particularly that occurring perimuscularly, appeared to be equally marked in the " Jectofer " rats and in the rats from the other groups.

*Tumour morphology*.—The fully developed tumours were round, firm bodies which over a period of 2 to 5 weeks grew to the size of an orange and often ulcerated the skin. On being transected they were found fairly well circumscribed from the surrounding muscles and adipose tissue. Centrally the larger ones invariably exhibited necrotic foci with cystic degeneration. Large retroperitoneal metastases were observed in two rats and pulmonary and pericardiac metastases were encountered in one rat. One of the metastatic tumours had been " Imferon " induced, the two others were " Ferrigen " induced.

---

### EXPLANATION OF PLATE

FIG. 6.—Accumulations of large, pigment-containing macrophages in perimuscular adipose tissue, 6 months after commencement of " Ferrigen " injections.

FIG. 7.—Intramuscular fibrosis and moderate nuclear polymorphism 8 months after commencement of " Ferrigen" injections.

FIG. 8.—Spindle cell sarcoma after " large dose " of " Imferon ".

FIG. 9.—Highly polymorphocellular sarcoma with " histiocytic " polynuclear giant cells induced by " large dose " of " Ferrigen ".

FIG. 10.—Muscular tissue infiltrated by predominantly spindle cell tumour tissue with abundant mitoses, induced by " large dose " of " Imferon ".

FIG. 11.—Moderately cell-rich fibroma induced by " Jectofer ".

Lundin.

At microscopic examination the tumours exhibited varying appearances. For the most part the picture was that of a low-differentiated tissue with an abundance of spindle cells, negligible collagen formation and fairly large numbers of mitoses (Fig. 8). Marginally the tumours infiltrated muscles and fat (Fig. 10). Occasional tumours were less rich in cells and distinctly collagen forming. Small areas with a definitely more pronounced cellular polymorphism could be observed in the majority of tumours. Some tumours exhibited a highly grotesque cellular polymorphism with large, often polynuclear cells containing giant nuclei and a profusely granulated cytoplasm, and large atypical mitoses (Fig. 9).

In the spindle celled tumours iron occurred predominantly in histiocytes having small nuclei and abundant cytoplasm which were interspersed among the tumour cells. Only minuscule quantities of iron were to be seen in the tumour cells themselves. Nevertheless, those tumours displaying the most accentuated cellular polymorphism invariably exhibited the presence of iron pigment also in these grotesquely polymorphic tumour cells.

The " Jectofer " induced tumour presented a deviating picture. Consistently very poor in cells, it exhibited small regular spindle cells without mitoses and lively collagen formation. Hence it distinctly differed from the " Ferrigen " and " Imferon " induced tumours and, on a purely histological basis, it should be classified more correctly as a fibroma (Fig. 11).

### DISCUSSION

These results verify previous workers' observations regarding the carcinogenic action of intramuscularly administered iron-dextran. Moreover, they indicate that an iron-dextrin complex exerts a similar action in principle. Richmond's (1959, 1960) and Haddow's (1959) assumptions that the carcinogenic action is bound to the iron are thus borne out. Just as in their experiments, our trials have failed to disclose any carcinogenic action of the isolated carbohydrate component (dextrin).

The correlation of tumour incidence to injected dose was not so obvious in the present investigation. Yet a statistically significant difference pointing in that direction was present between the groups treated with large and small " Ferrigen " doses. In addition there was a tendency to later tumour onset in the groups receiving small " Ferrigen " and " Imferon " doses than in the corresponding high-dosage groups.

When the incidence of tumours is related to the iron concentration at the site of the injection, it appears that the number of tumours was definitely lower in the small-dose " Ferrigen " group than in the small-dose " Imferon " group, notwithstanding the fact that the local iron concentration of the muscles was higher in the former group. In addition, the rats from the " Ferrigen " groups displayed a later tumour onset than those from groups with corresponding dosages of " Imferon ".

The local iron concentration in the muscles of the " Jectofer " treated rats was clearly lower than in the rats treated with either high or low doses of " Ferrigen " and " Imferon ". Herein may lie the reason for the absence of sarcomas in the former group. Another possible prerequisite for the nondevelopment of sarcomas might be the considerably less marked histiocytic reaction which, at least to some extent, would seem to be a consequence of the low molecular weight of " Jectofer ".

Needless to say, the development of a fibroma-like tumour in this group must have been brought about by the injections.

*Histogenesis.*—In Richmond's opinion the tumours were most nearly classifiable as histiocytomas and he apparently believed them to arise from those histiocytes charged with iron pigment which proliferate in the muscle septa and perimuscular connective tissue. He also demonstrated that the polymorphic giant cells encountered in some of these tumours are susceptible to silver impregnation by Marshall's modification of Weil-Davenport's method. This may indicate that such cells possess histiocytic characteristics. Curiously enough, however, in the majority of tumours iron pigment is rather sparse in the tumour tissue itself, and by far the greater part of any pigment present is found in morphologically typical macrophages lacking signs of cellular polymorphism. On the other hand, large amounts of iron pigment are often encountered in the polymorphic giant cells.

In my experience, however, any cellular polymorphism seen in the " premalignant " phase can be observed most distinctly in the fibrosis which is particularly marked perimuscularly. Fibroblast-like, often polynuclear cellular elements with irregular large nuclei and also some mitoses were encountered in such regions some 6 to 9 months after the commencement of the experiment. The histiocytic elements seemed to exhibit a less conspicuous cellular polymorphism which, however, was more difficult to judge owing to the abundance of pigment present in these cells.

Accordingly the most prevalent tumour tissue component with densely packed spindle cells should perhaps be interpreted as fibrosarcoma. Everything suggests, however, that the designation histiocytic sarcoma is deserved by the highly polymorphic tumours with giant cells which often contain iron pigment and, according to Richmond, are positive to Marshall's silver impregnation.

The collagen fibrils in the muscle septa and vascular walls at an early stage consistently showed a uniformly intense, positive iron reaction. Consequently the carcinogenic action of iron can be exerted not only against the histiocytic cells but no doubt also against the fibroblasts.

*Mode of action of iron.*—Since the carcinogenic action of iron can be induced with the iron bound in different complexes, the iron component seems responsible for this action. Conceivably the other component is essential merely as a mediator facilitating the deposition of enough iron in the tissues and enabling it to be retained and slowly absorbed so that a high and constant iron concentration is maintained. Richmond expressed the view that the dextran component made it easier for the iron to enter the cell. The histiocytic reaction is surely dependent upon molecular size, and therefore upon molecular weight, and is unlikely to occur below a given minimum molecular size. In conformity with this we have found that " Jectofer "—whose molecular weight is much smaller than that of either " Ferrigen " or " Imferon "—induces a distinctly less marked histiocytic reaction by virtue of its smaller molecules than either of the other iron complexes tested.

In view of the high incidence of tumours in the muscles, it is a remarkable thing that tumours had not developed in the liver or spleen before the experiment was discontinued. The concentration of iron, especially in the " Imferon " group, was of the same order in the liver as in the muscles. Notably, however, the iron content of the liver was histologically found mainly in groups of very large macrophages the majority of which were located periportally, there being no appreciable amounts of iron deposited in the collagen, stroma or vascular walls. Nor did we observe any proliferation of hepatic connective tissue, thus verifying the reports of Golberg *et al.* (1960) Yet one would expect the chances for the development of histiocytic tumours in the liver to be as great as at the site of the injection. Conversely, if most of the tumours are interpreted as being of fibrosarcomatous type, then the absence of fibrosis in the liver can well be reconciled with the absence of carcinogenic activity there. Haddow and Horning (1960) observed occasional hepatomas but reported no sarcomas or histiocytomas in the liver.

The mechanism of the carcinogenic action exerted by iron is of course just as obscure as that exerted by other carcinogenic substances. Richmond and Had dow discussed a variety of conceivable points of interference with cellular function, such as disturbed nucleic acid, vitamin E and haemoglobin metabolism or upsets in various enzymatic systems. Hueper (1957) postulated that the formation of macromolecular protein carcinogens might be a common denominator for the action cf different types of carcinogenic substances. It is remarkable that Hueper also got tumours (in most cases malignant lymphomas) after injection in mice of high molecular weight dextran of different degree of branching of the molecule (Hueper, 1959).

SUMMARY

The carcinogenic actions of three different iron complexes intramuscularly injected into rats have been studied. Both " Imferon " (iron dextran) and " Ferrigen " (iron dextrin) induced a high incidence of sarcomas 9 to 14 months after commencement of the injections, whereas " Jectofer " (iron-sorbitol-citric acid) after the same length of time induced but a single tumour with the histological features of a fibroma. The results are discussed in the light of, among other factors, the amount of iron deposited and the differing molecular weights of the substances.

### REFERENCES

BEUTLER, E., ROBSON, M. J. AND BUTTERWIESER, E.—(1958) *Ann. intern. Med.*, **48**, 60.
ERIKSSON, A. F. V., to be published 1962.
FAULDS, J.—(1954) *Int. Congr. clin. Path.*, Washington (cited from Heuper 1957)
GOLBERG, L., MARTIN, L. E. AND SMITH, J. P.—(1960) *Toxicol. appl. Pharmacol.*, **2**, 683.
HADDOW, A.—(1959) *In* ' Ciba Foundation Symposium on Carcinogenesis '. London (Churchill), p. 300.
*Idem* AND HORNING, E. S.—(1960) *J. nat. Cancer Inst.*, **24**, 109.
HUEPER, W. C.—(1959) *Arch Path.* (*Lab. Med.*), **67**, 589.—(1957) *In* ' Cancer ', Ed. C. E. Raven. London (Butterworth & Co.), Vol. 1.
KENNAWAY, N. M. AND KENNAWAY, E. L.—(1936) *J. Hyg.*, *Camb.*, **36**, 236.
RICHMOND, H. G.—(1959) *Brit. med. J.*, i, 947.—(1960) *In* ' Cancer Progress '. London (Butterworth & Co.), p. 24.